# Supplementary material for: Modeling genome-wide enzyme evolution predicts strong epistasis underlying catalytic turnover rates
Source: Nat Commun. 2018 Dec 10;9:5270. doi: 10.1038/s41467-018-07649-1 (PMC6288127; doi:10.1038/s41467-018-07649-1)
Supplement: Supplementary file 1 — Supplementary Information [file 41467_2018_7649_MOESM1_ESM.pdf]

Supplementary Information

Modeling genome-wide enzyme evolution predicts strong epistasis underlying catalytic turnover rates

Heckmann et al.

Supplementary Table 1: Reactions connected to “jumps” in fitness trajectories. These reactions were selected as the top 0.1% in terms of absolute fitness gain.

bigg_id	Freq	description	GPR
3OAR121	1	3-oxoacyl-[acyl-carrier-protein] reductase (n-C12:1)	b1093
3OAR141	2	3-oxoacyl-[acyl-carrier-protein] reductase (n-C14:1)	b1093
3OAR161	1	3-oxoacyl-[acyl-carrier-protein] reductase (n-C16:1)	b1093
3OAR40	1	3-oxoacyl-[acyl-carrier-protein] reductase (n-C4:0)	b1093
AICART	2	Phosphoribosylaminoimidazolecarboxamide formyltransferase	b4006
G1PACT	2	Glucosamine-1-phosphate N-acetyltransferase	b3730
HISTP	3	Histidinol-phosphatase	b2022
IGPDH	6	Imidazoleglycerol-phosphate dehydratase	b2022
IMPC	1	IMP cyclohydrolase	b4006
MOAT2	1	3-deoxy-D-manno-octulosonic acid transferase	b3633

Supplementary Table 2: Sensitivity of correlation between experimental data and model predictions against identity and size of the unconstrained (evolving) set of reactions. A random set of unconstrained reactions of size 373 (equal to the size used for simulation presented in Figure 4), 750, and 1000 was set. The average evolved k_{cat} vector across six replicates per set size was compared to experimental data. Aerobic growth on glucose was used as growth condition. Reactions unchanged after simulation under this constant condition were removed when evolved k_{cat} was below 10^{-2}. Note that the number of comparable observations are significantly lower than in the original analysis presented in Figure 4 because of the random nature of the evolving reaction set. The *p*-values were calculated as described in in the Methods section.

**number of randomly chosen evolving reactions**	**Pearson's *R***	***p* value**	**number of observations**	**experimental data source**	**number of replicates**
373	0.5909765	1.66E-11	106	in vivo kapp,max	6
750	0.63616451	4.51E-19	154	in vivo kapp,max	6
1000	0.69195051	7.74E-26	170	in vivo kapp,max	6
373	0.33494898	0.03713	37	in vitro kcat	6
750	0.49173083	0.000103	55	in vitro kcat	6
1000	0.49711089	3.95E-05	60	in vitro kcat	6

Supplementary Table 3: Sensitivity of correlation between experimental data and model predictions against the ratio of beneficial mutations to deleterious mutations. The average evolved k_{cat} vector across three replicates per set size was compared to experimental data. Aerobic growth on glucose was used as growth condition (as in Supplementary Figure 11, where the ratio is 100). We find that the ability of the

model to explain experimental data is robust to changes in the assumed frequency of deleterious mutations. The *p*-values were calculated as described in in the Methods section.

**frequency ratio of deleterious to beneficial mutations**	**Pearson's *R***	***p* value**	**number of observations**	**experimental data source**	**number of replicates**
10	0.559928	8.25E-19	209	in vivo kapp,max	3
100	0.569249	1.62E-19	209	in vivo kapp,max	3
1000	0.584914	9.26E-21	209	in vivo kapp,max	3
10	0.294101	0.012156	70	in vitro kcat	3
100	0.311205	0.007794	70	in vitro kcat	3
1000	0.410868	0.000337	70	in vitro kcat	3

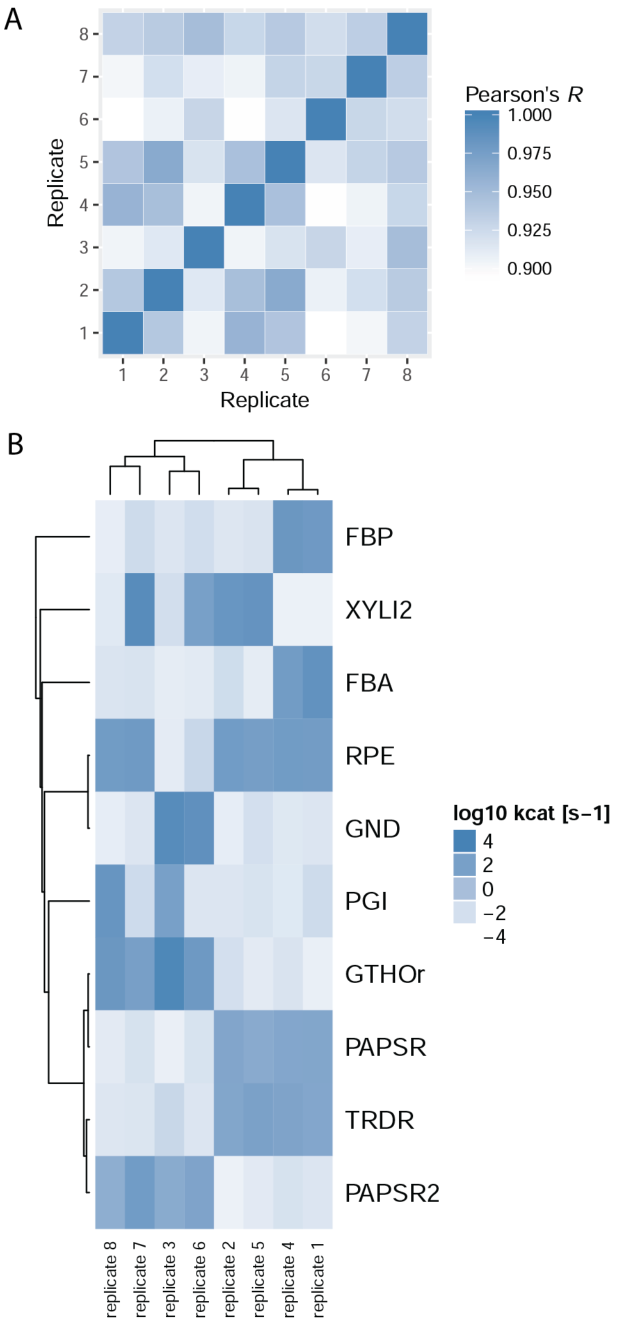

Supplementary Figure 1: Most evolved k_{cat}s show a high level of convergence. (A) The correlation (Pearson's R) between k_{cat}s that are not subject to biophysical constraints is shown. (B) Examples of divergent k_{cat}s were selected as reactions with the highest standard deviations in k_{cat} across replicates. Single linkage clustering shows reactions for which high k_{cat}s are mutually exclusive across replicates.

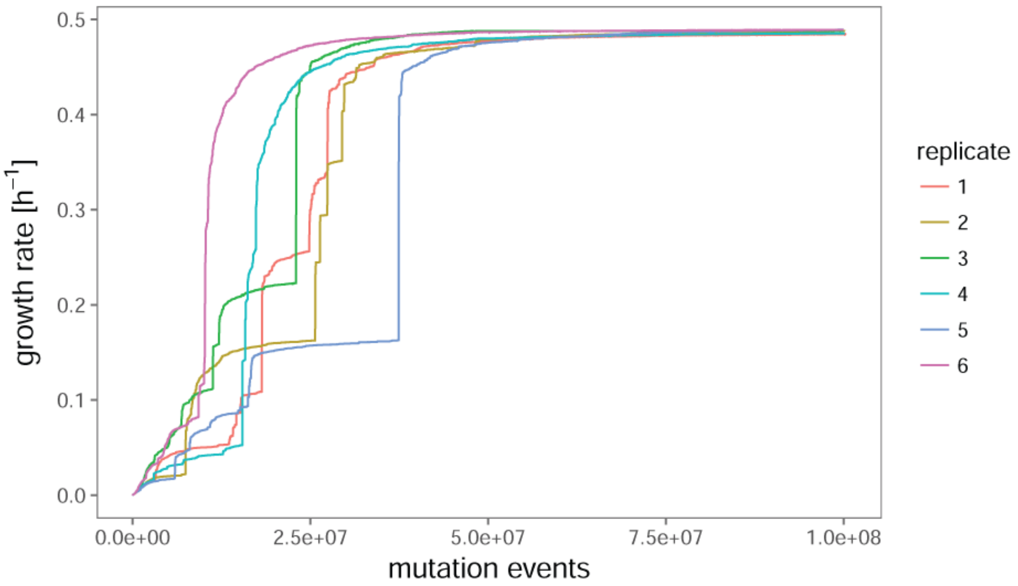

Supplementary Figure 2: Continued evolutionary trajectories on aerobic glucose. Related to Figure 2. The growth rate of the population is plotted against the number of simulated mutations.

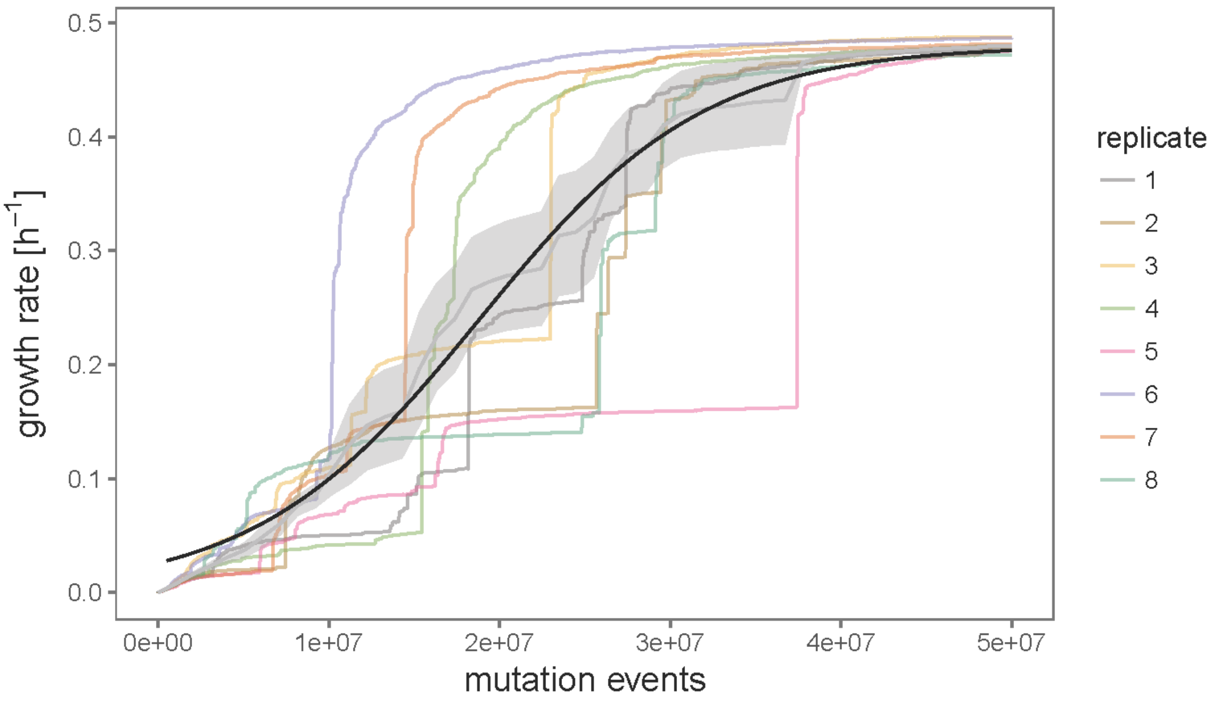

Supplementary Figure 3: The growth rate of the population against the number of simulated mutations, as presented in Figure 2A, with a fitted simple mechanistic model of k_{cat} evolution as presented in Supplementary Equation 3. Least squares minimization was conducted using the Nelder-Mead algorithm as implemented in the optim() function of the R environment. Fitted parameters were: C=0.48, $(p/q)/(k_{cat2}/k^{0}_{cat1})$=17.43, α=1+1.52e-7. Note that, while the general adaptation dynamic is captured by the simple model, the effect of initial mutation events is not described well.

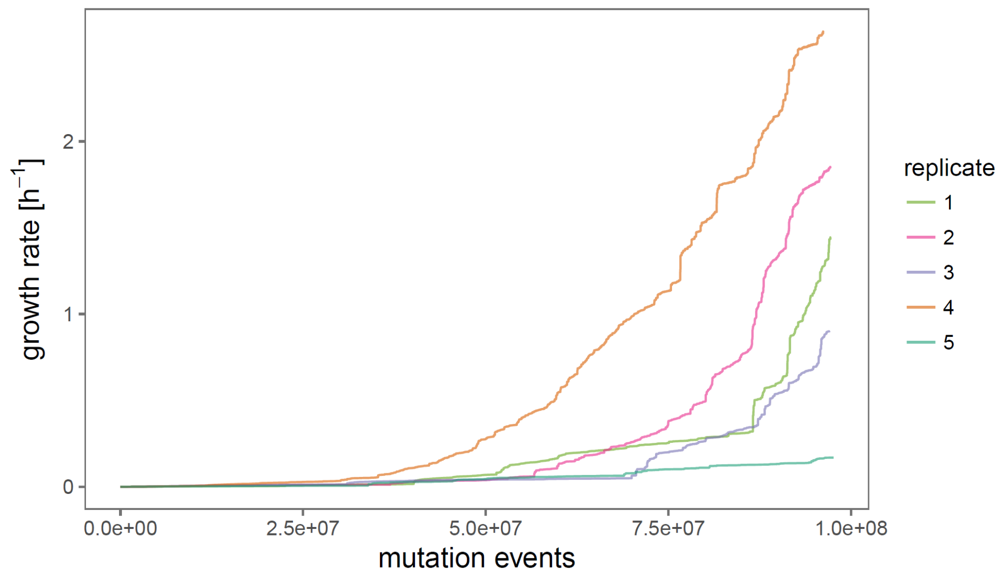

Supplementary Figure 4: Simulated evolutionary trajectories when no biophysical constraints on any k_{cat}s are applied. Related to Figure 2. Growth rates increase exponentially and exceed measured aerobic growth rates for *E. coli* on glucose minimal medium (~1.0 h^{-1},[1]).

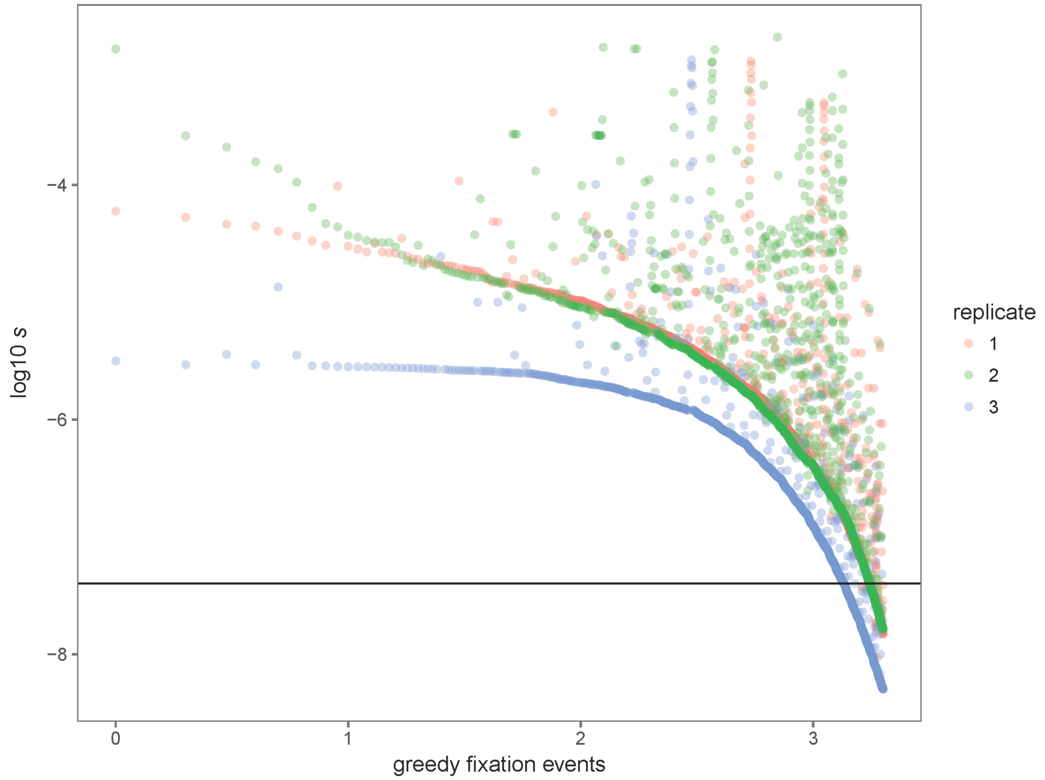

Supplementary Figure 5: Greedy continuation of evolutionary end points shown in Supplementary Figure 2. As fixation probabilities diminish, MCMC becomes computationally infeasible for tracing the later stages of evolution. In order to investigate the effect of additional mutations computationally, a greedy simulation approach is used. At each iteration, we compute the selection coefficient *s* for an individual doubling (i.e., α=2) of all unconstrained k_{cat}s. The "mutation" that yields the highest *s* is fixed and the algorithm is repeated. Two thousand of those fixation events are shown for each replicate. The black line indicates the theoretical limit for s below which mutations behave effectively neutral. The final growth rates after iteration 2000 are: 0.492 h^{-1}, 0.496 h^{-1}, and 0.497 h^{-1}, for replicate 1, 2, and 3, respectively.

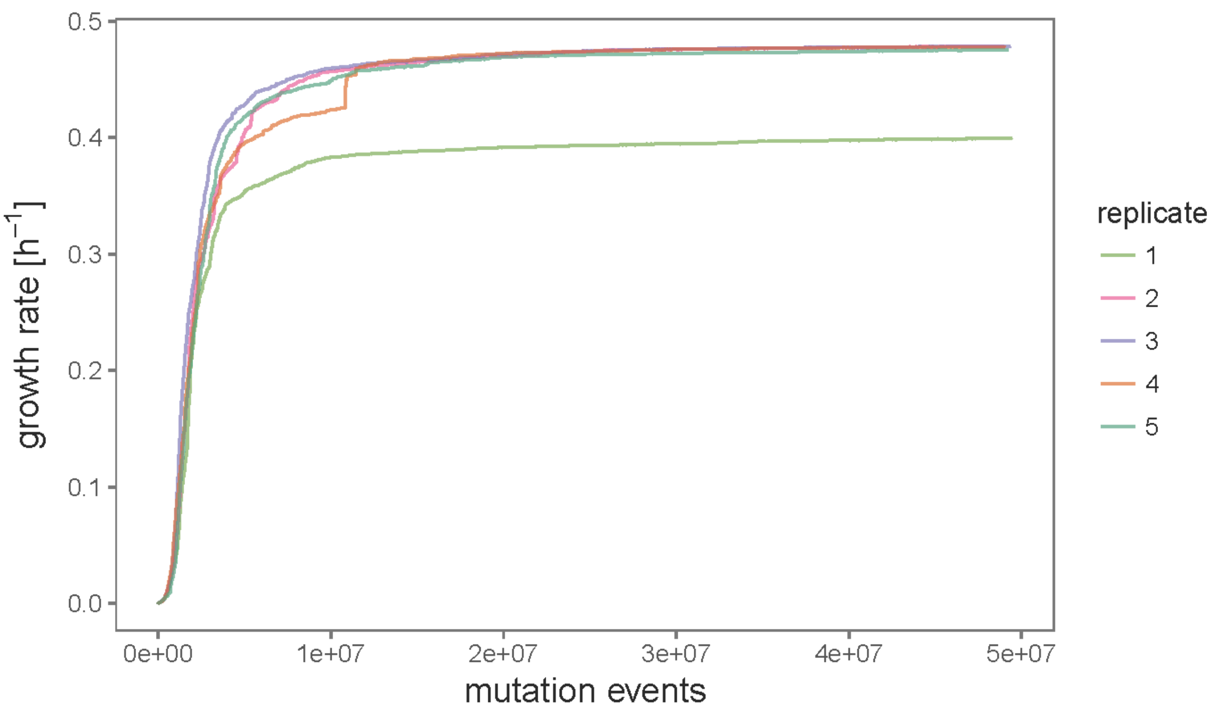

Supplementary Figure 6: Constraining multi-functional genes abolishes jump dynamics. All reactions catalyzed by genes that were associated with "jumps" in adaptation trajectories (as listed in Supplementary Table 1) were added to the set of constrained reactions. The correlation with experimental data at the end points is R=0.23 (p<0.052) for in vitro data and R=0.53 (p<3.5e-14) for $k_{app,max}$ (compare to Supplementary Figure 10).

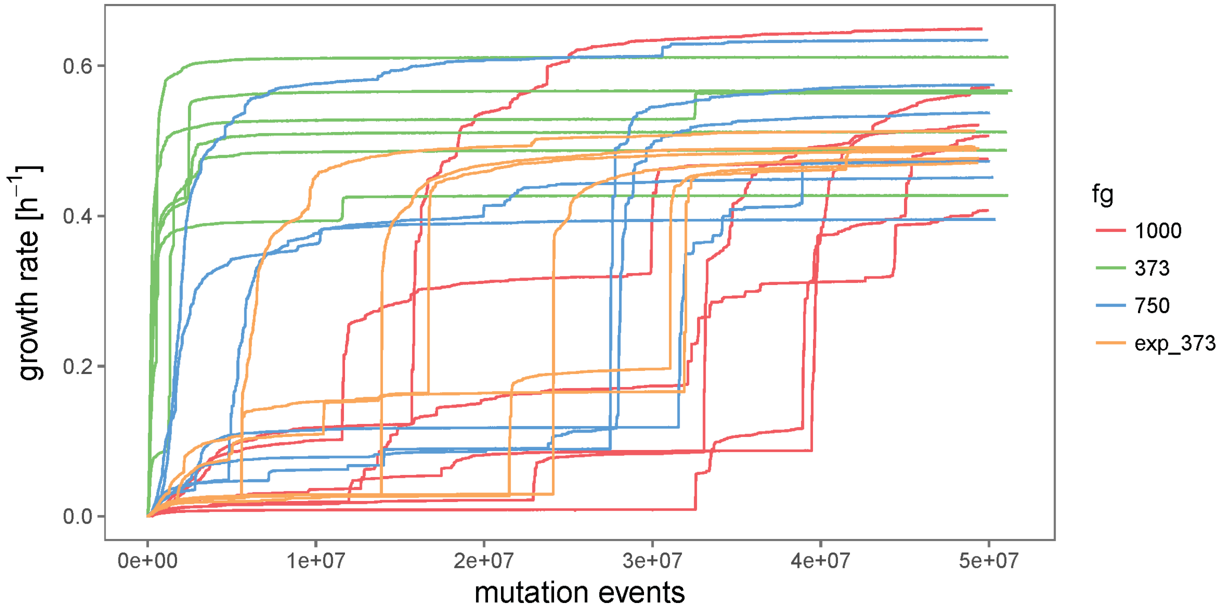

Supplementary Figure 7: Sensitivity analysis against identity and size of the set of evolving reactions. Growth rate trajectories of k_{cat} evolution are shown for a randomly chosen set of unconstrained evolving

reactions of size 1000, 750, 373 (the number of reactions for which experimental data is available), and an unconstrained set that consists of the 373 reactions for which experimental data was available (“exp_373”). Differences in final growth rates across set sizes are not significant (p>0.76, ANOVA), although this result might be affected by differences in speed of adaptation.

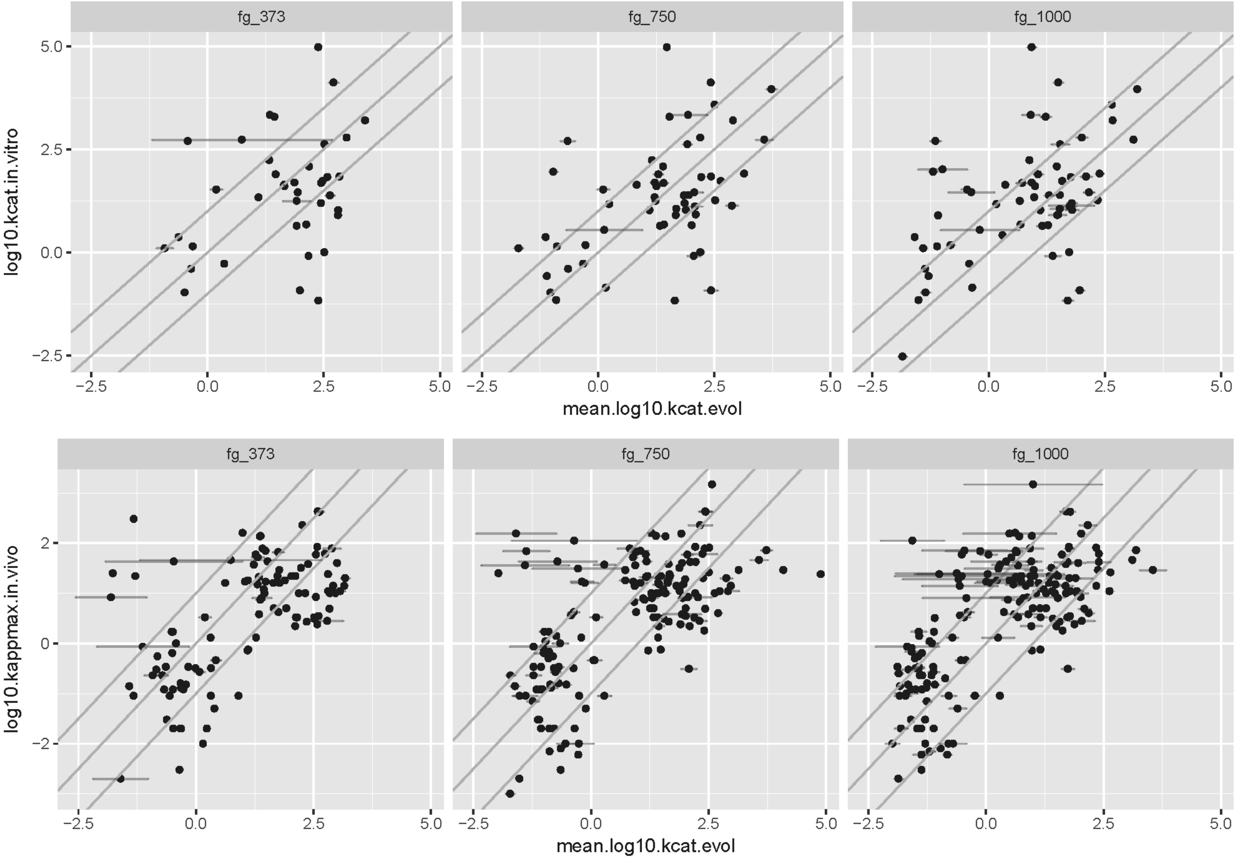

Supplementary Figure 8: Sensitivity of correlation between experimental data and model predictions against identity and size of the unconstrained (evolving) set of reactions. A random set of unconstrained reactions of size 373 (fg_373, equal to the size used for simulation presented in Figure 4), 750 (fg_750), and 1000 (fg_1000) was set to simulate k_{cat} evolution across 6 replicates per condition. Predictions are compared to experimental data, where horizontal bars show standard errors across replicates. See Supplementary Table 2 for statistics and details.

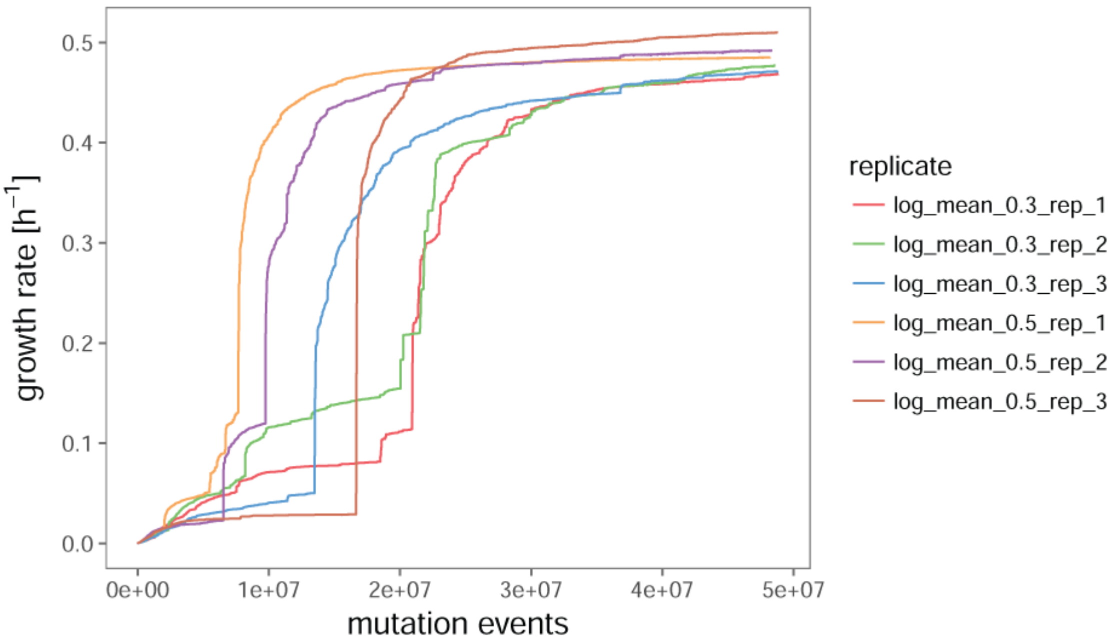

Supplementary Figure 9: Sensitivity analysis for size of mutational effects. Growth rate trajectories of k_{cat} evolution are shown for different average mutation effect sizes. The original mean of the log-normal distribution of mutation effects in log scale (log(3/2)≈0.4, see Methods) was increased to 0.5 in three replicates, and decreased to 0.3 in three additional replicates. The correlation of k_{cat} vectors averaged over replicates with *in vitro* data is R=0.24 (p<0.028) for log(mean)=0.3 and R=0.25 (p<0.021) for log(mean)=0.5. For $k_{app,max}$, R=0.55 (p<2e-18) for log(mean)=0.3 and R=0.55 (p<2e-18) for log(mean)=0.5. The p-values were calculated as described in in the Methods section.

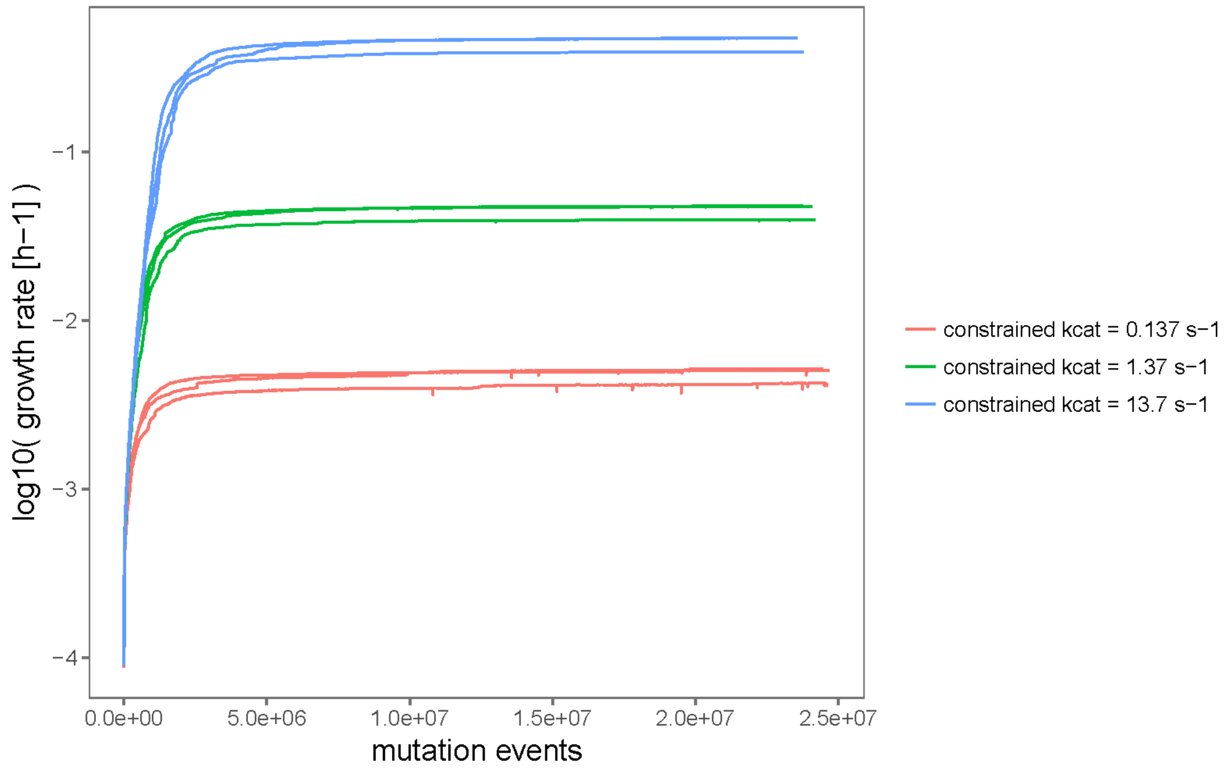

Supplementary Figure 10: Magnitude of biochemically constrained k_{cat}s determines final growth rates. To determine the effect of the magnitude of constrained k_{cat}s on end point growth rates, we simulate three replicates each for the original case (all constrained k_{cat}s set to 13.7s^{-1}) and for two reduced cases (1.37s^{-1} and 0.137s^{-1}). To speed up convergence and reduce variance across replicates we removed multifunctional reactions as explained in Supplementary Figure 6. We find that the k_{cat} of the constrained fraction is a major determinant of the final growth rate.

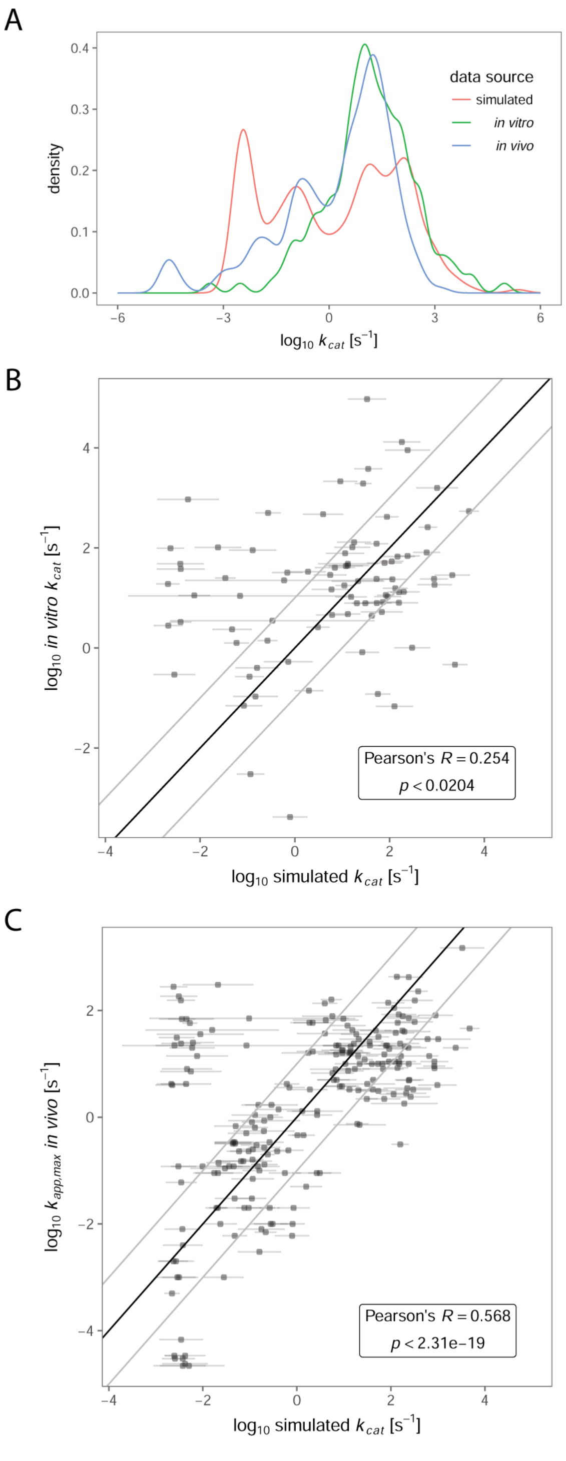

Supplementary Figure 11: Comparison between k_{cat} predictions for evolution on aerobic glucose and experimental data. (A) Distribution of turnover rates in *in vitro*, *in vivo*, and simulated data. Simulated data is only shown for non-constrained reactions that contribute to growth. (B, C) Comparison between experimental and simulated data. Horizontal error bars show the standard deviation across simulated replicates. The *p*-values were calculated as described in in the Methods section.

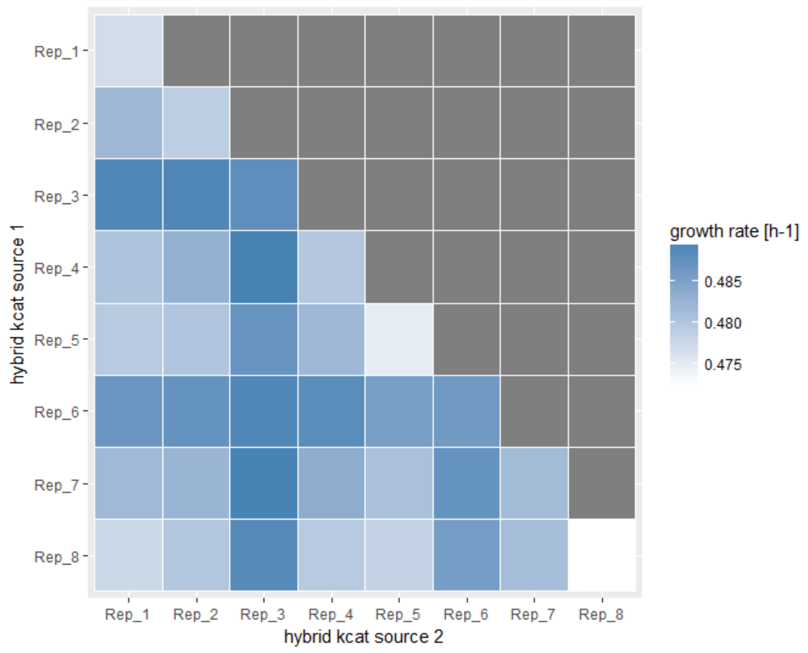

Supplementary Figure 12: Growth rate of combining evolutionary end points as presented in Figure 2. The convergence of replicates in terms of growth rate and k_{cat} suggests a smooth, single-peaked phenotypic fitness landscape. In order to investigate the structure of the fitness landscape between simulated end-points, we average k_{cat} vectors between pairs of replicates and compute the corresponding growth rate. This “hybridization” indicates that no “fitness valleys” exists between end points, supporting the idea of a single-peaked landscape.

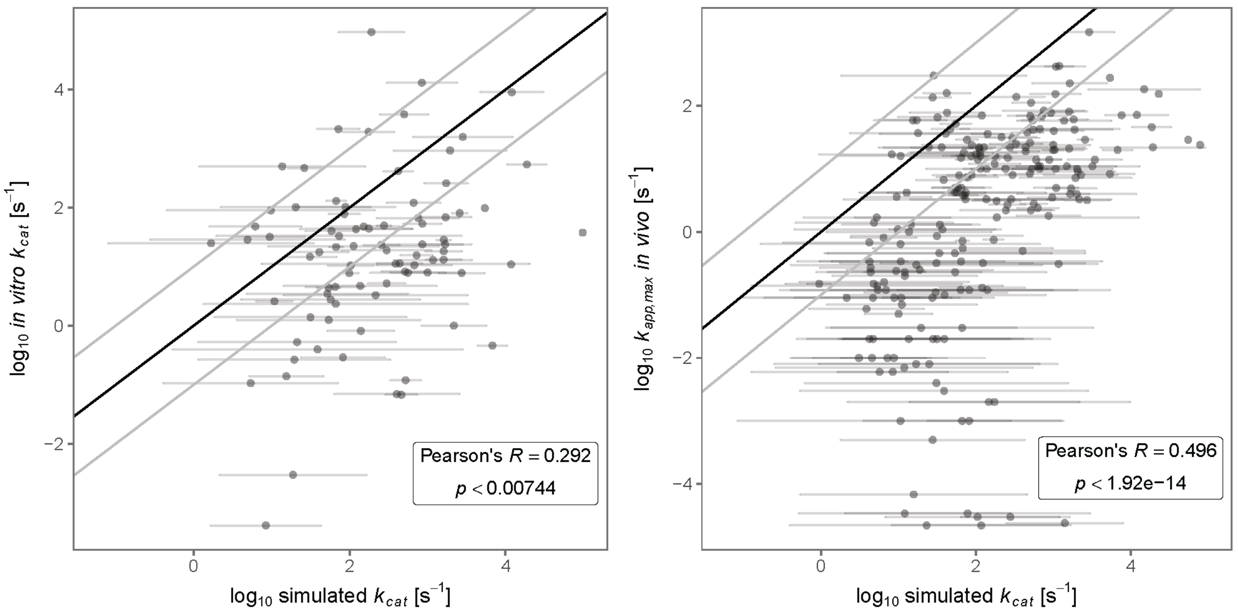

Supplementary Figure 13: Comparison between experimental data and end point k_{cat} predictions from simulations where initial states are sampled from an empirical distribution. Initial k_{cat}s of five replicates were drawn from a log-normal distribution with log-scaled mean 19.12s^{-1} and log-scaled standard

deviation 18.88s^{-1}, as determined from the *in vitro* data in [2] that could be mapped to the model. To avoid numerical problems in the simulation, values were capped below 1e-3 and above 1e5. Lower correlation than in the original simulations (Figure 4) and over-estimation of experimental data is found. The likely cause for this decreased agreement with experimental data are unrealistically high k_{cat}s in initial states, that then have a low probability of deterioration because, even in un-used reactions, decreases in k_{cat}s act at best as neutral mutations. Horizontal error bars in show the standard deviation across five simulated replicates. The *p*-values were calculated as described in in the Methods section.

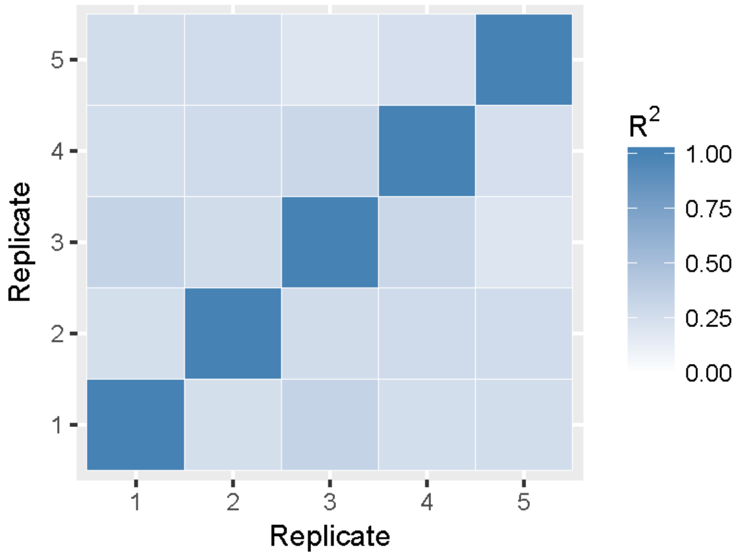

Supplementary Figure 14: Comparison of end point k_{cat} vectors when initial states are sampled from an empirical distribution (see Supplementary Figure 12 for details).

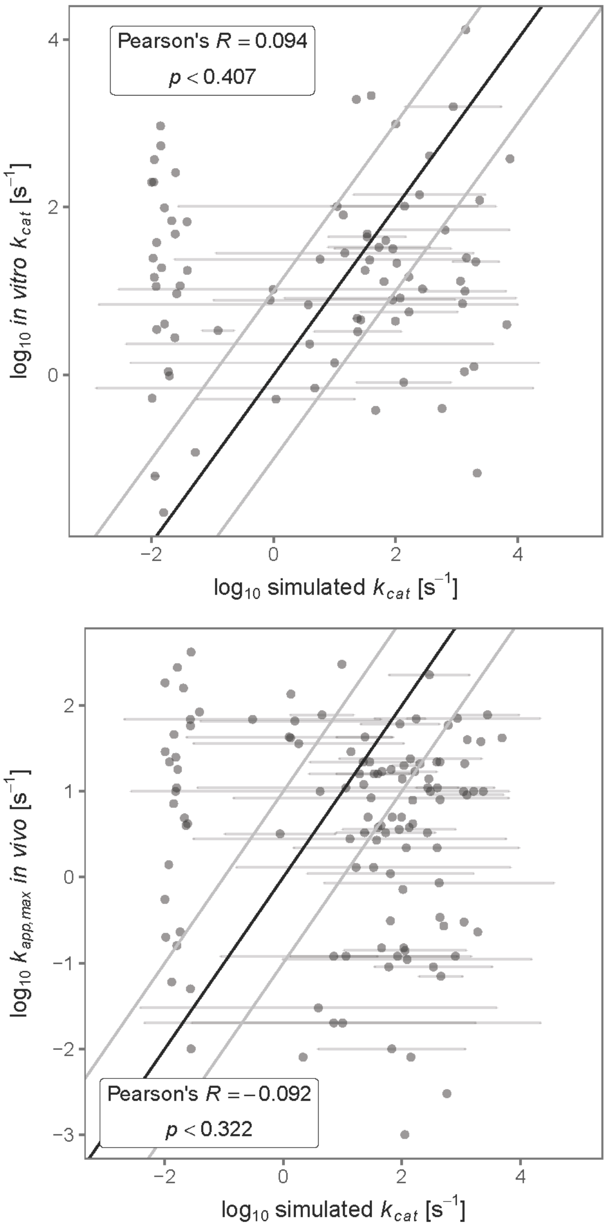

Supplementary Figure 15: Random perturbation of network stoichiometry and biomass equation abolishes correlation with experimental data. In order to test the effect of network structure and flux distribution on the ability of the model to explain experimental data, we replace stoichiometric coefficients in the metabolic model iJO1366 with integers drawn from a binomial distribution with five trials of success probability ½, where the original sign of the coefficients remains intact. Furthermore, we perturb the flux distribution of the model by replacing the original biomass function of the model with a random sample of consumed components of the same cardinality as the original biomass function (68 metabolites), and with coefficients mimicking the distribution of the original biomass function of the model (normal distribution with mean 1.60 and standard deviation 8.74). This process was repeated until a model with feasible growth was found. Protein molecular weights were set to the median of the model enzymes (=44kDa). k_{cat} evolution was simulated analogously to that presented in Figure 2, i.e., 5e7 mutations for aerobic growth on glucose were simulated for five replicates with different reaction

stoichiometries and biomass functions. Points show averages over these replicates, with error bars showing standard deviations in case more than one replicate showed significant evolution for that reaction. The *p*-values were calculated as described in in the Methods section.

Supplementary Note 1: A simple model for the emergence of diminishing returns in k_{cat} evolution

A model of multiplicative mutations

Diminishing returns are a prominent feature of the evolutionary trajectories that are predicted by our genome-scale approach. Here, we analyze the effect of biophysical constraints on k_{cat} evolution in a simple model of two reactions with the aim of elucidating the mechanisms that cause diminishing returns and to test the effect of different model assumptions.

In this simple model, each reaction is catalyzed by a single gene product, where one gene can evolve indefinitely, while the other is subject to a biophysical constraint that does not allow for further improvements in k_{cat}. Furthermore, the two enzymes act in a linear and fitness-relevant pathway, and the respective activities v_1 and v_2 [mol s-1 gDW-1] are calculated analogously to the genome-scale approach (Supplementary Fig. 16).

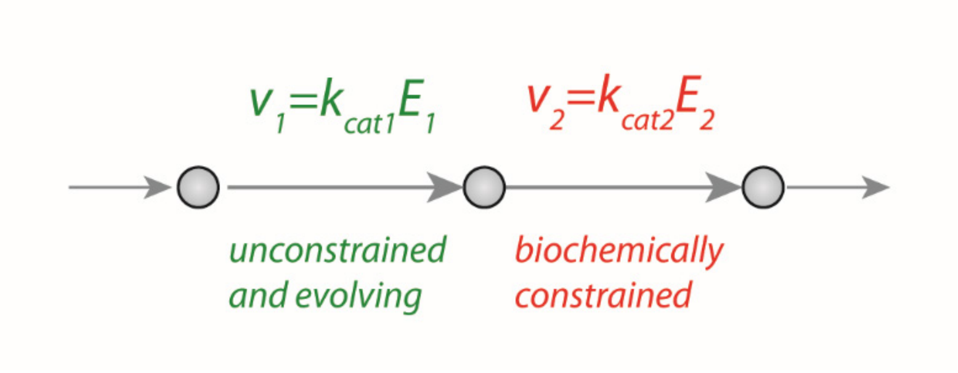

Supplementary Figure 16: A simple two-reaction model with one evolving (E_1 [mol gDW^{-1}], green) and one biochemically constrained (E_2 [mol gDW^{-1}], red) reaction.

The genome-scale simulation assumes optimal gene expression patterns during the adaptation process. We can impose this assumption by applying a uniform distribution of activities as

$$v_1 = v_2 = k_{cat1}E_1 = k_{cat2}E_2.$$

Furthermore, a proteome constraint C [mol gDW-1] is applied as

$$E_1 + E_2 = C.$$

The value of k_{cat1} [s-1] after n mutations of equal multiplicative effect α is

$$k_{cat1}^{n} = \alpha^{n} k_{cat1}^{0},$$

where k^{0}_{cat1} represents the ancestral turnover number.

Combining these equations and re-arranging yields

$$E_2 = \frac{C}{\alpha^{-n}\frac{k_{cat2}}{k_{cat1}^{0}} + 1}. \qquad (1)$$

As k_{cat2} is constant, E_2 is proportional to the systems fitness. When mutations acting on k_{cat1} are beneficial (α>1), the development of the evolving system with increasing number of mutations takes a sigmoidal shape (Supplementary Fig. 17).

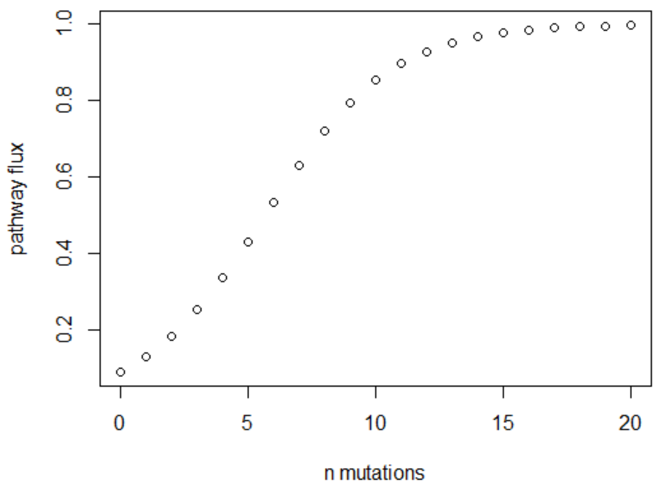

Supplementary Figure 17: Pathway flux as given when mutations act multiplicatively (Supplementary Equation 1). Parameters used are: C=1, k_{cat2}/ k^{0}_{cat1}=10, α=3/2.

Also note that with increasing improvement of k_{cat1} (α>1), the proteome investment into the biophysically constrained second reaction approaches the total proteome C:

$$\lim_{n\to\infty} E_2 = C.$$

Because of the finite population size in real organisms, E_2 will not approach C indefinitely; when the fitness gain is too low dynamics become dominated by random drift[3,4].

A model of additive mutations

We further analyze a similar model, where mutations act on k_{cat1} in an additive manner;

$$k^{n}_{cat1,add} = n\beta + k^{0}_{cat1}.$$

This leads to the following expression for E_2:

$$E_{2,add} = \frac{C(n\beta + k^{0}_{cat1})}{k^{0}_{cat1} + n\beta + k_{cat2}}. \quad (2)$$

Again, this additive model shows saturation and diminishing returns (Supplementary Fig. 18).

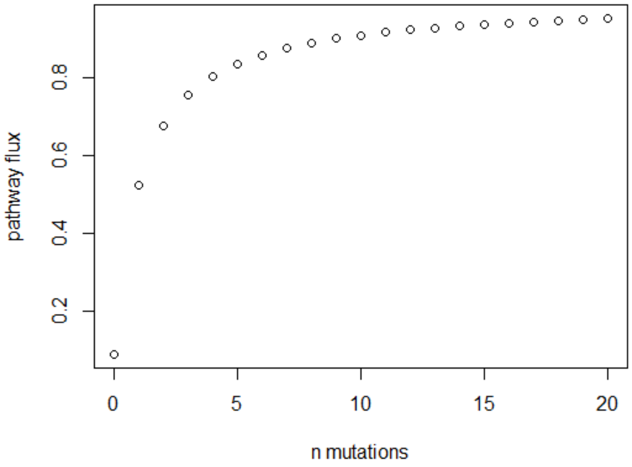

Supplementary Figure 18: Pathway flux as given when mutations act additively (Supplementary Equation 2). Parameters used are: C=1, k^{0}_{cat1}=1/10, k_{cat2}=1, β=1.

Notice that, again, $E_{2,\mathrm{add}}$ approaches C as mutations accumulate:

$$\lim_{n\to\infty} E_{2,add} = C.$$

The effect of the number of evolving reactions

To extend the multiplicative model with multiple reactions that act in a linear pathway, we make the simplifying assumption that p evolving reactions share a common k_{cat}, k^{0}_{cat1}, while the constrained set of q reactions shares k_{cat2}:

$$E_{evolving} + E_{constrained} = C.$$

$E_{evolving}$ denotes the proteome fraction in evolving enzymes, while $E_{constrained}$ represents the biophysically constrained fraction. Uniform distribution of enzyme activity is applied as:

$$\frac{E_{evolving}}{p} k_{cat1} = \frac{E_{constrained}}{q} k_{cat2}.$$

The protein investment in the constrained fraction is then given by:

$$E_{constrained} = qE_2 = \frac{C}{\frac{p}{q}\alpha^{-n}\frac{k_{cat2}}{k_{cat1}^0} + 1}. \quad (3)$$

As expected, diminishing returns are still occurring, and the total achievable flux of the pathway decreases with the number of constrained reactions (Supplementary Fig. 19).

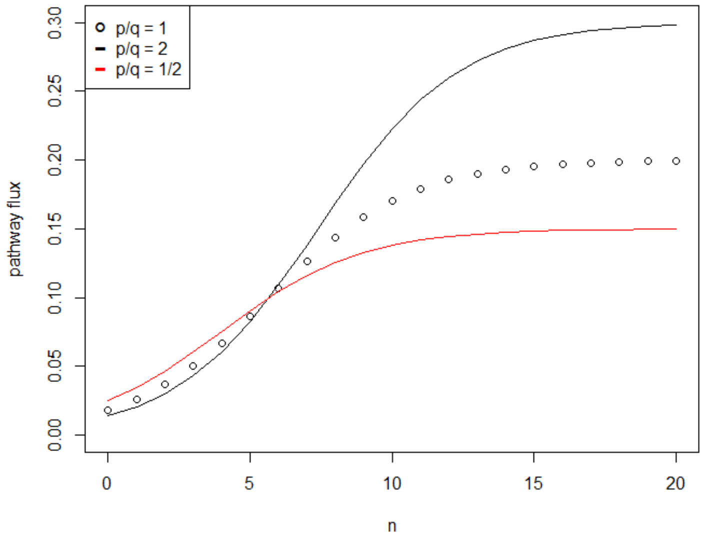

Supplementary Figure 19: Pathway flux as given when mutations act multiplicatively on multiple evolving and constrained reactions (Supplementary Equation 3). Parameters used are: C=1, k_{cat2}/k^0_{cat1}=10, α=3/2. The ratios of evolving to constrained reactions are 1 (dots), 2 (black line) and ½ (red line). The total number of reactions is 10=p+q.

The limiting case is now a function of the number of constrained reactions:

$$\lim_{n\to\infty} E_2 = \frac{C}{q}.$$

This confirms our intuition: the achievable fitness decreases as the fraction of constrained reactions grows.

A fit of Supplementary Equation 3 to simulated trajectories is shown in Supplementary Figure 3.

Determinants of the selection coefficient s

We next determine the selection coefficient s for a novel mutation that changes the k_{cat} of one reaction of the evolving set by a factor α.

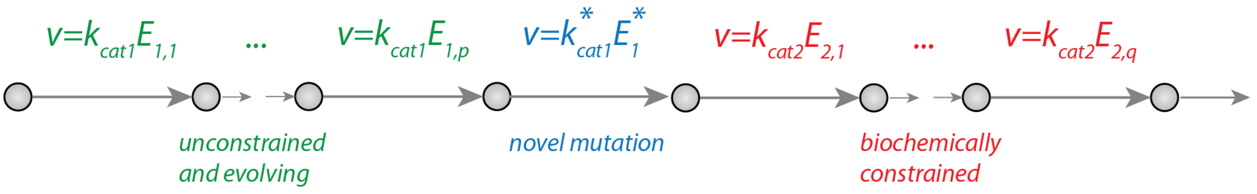

Supplementary Figure 20: Modelling the effect of a single novel mutation on pathway flux.

Following the approach above for a linear pathway of M reactions with q constrained reactions (Supplementary Fig. 20), we arrive at the following equation for s:

$$s = 1 - \frac{\frac{k_{cat2}}{k_{cat1}}\left(\frac{1}{\alpha} + (M-q-1)\right) + q}{\frac{k_{cat2}}{k_{cat1}}(M-q) + q} = \frac{(\alpha-1)k_{cat2}}{\alpha(k_{cat1}q + k_{cat2}(M-q))}$$

This result shows that the adaptive advantage of a mutation that increases the k_{cat} of one reaction by α, decreases drastically with the size of the system under selection (M) (Supplementary Fig. 21).

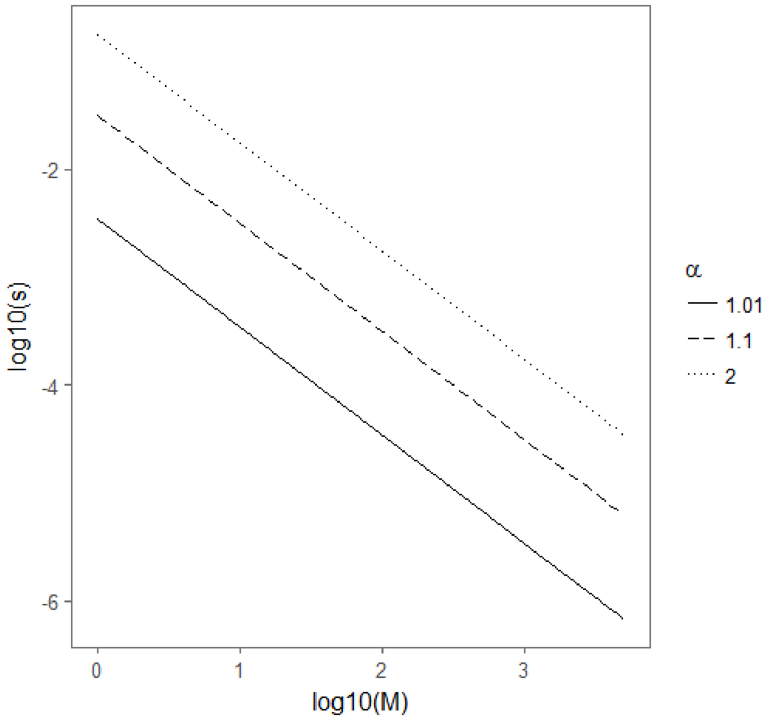

Supplementary Figure 21: The effect of number of reactions (*M*) on the selection coefficient (*s*) for a mutation of magnitude α in the kcat of a single reaction. The ratio between k_{cat2} and k_{cat1} was 0.05, *p* and *q* were chosen to have 10% of reactions in the biochemically constrained set.

Our simulation results for the metabolic network of *E. coli* indicate that diminishing returns render the selective advantage of mutations that affect individual k_{cat}s so low that mutations act as essentially neutral long before a potential optimum of the system is reached (see main Text). We find a similar behavior for the simple model of the linear pathway, where the scenario shown in Supplementary Figure 21 exhibits a total pathway flux that is 69% of the flux the system would exhibit if evolving reactions had reached a k_{cat} of 1e5. The size of the network is thus a strong determinant of whether natural selection can drive the system closer to the theoretical optimum.

Supplementary References

1 LaCroix, R. A. *et al.* Use of Adaptive Laboratory Evolution To Discover Key Mutations Enabling Rapid Growth of *Escherichia coli* K-12 MG1655 on Glucose Minimal Medium. *Appl Environ Microb* **81**, 17-30, doi:10.1128/Aem.02246-14 (2015).

2 Bar-Even, A. *et al.* The Moderately Efficient Enzyme: Evolutionary and Physicochemical Trends Shaping Enzyme Parameters. *Biochemistry* **50**, 4402-4410, doi:10.1021/bi2002289 (2011).

3 Li, W. H. Maintenance of Genetic-Variability under Joint Effect of Mutation, Selection and Random Drift. *Genetics* **90**, 349-382 (1978).

4 Kimura, M. *The neutral theory of molecular evolution*. (Cambridge University Press, 1983).
