# Supplementary material for: Modeling genome-wide enzyme evolution predicts strong epistasis underlying catalytic turnover rates
Source: Nat Commun. 2018 Dec 10;9:5270. doi: 10.1038/s41467-018-07649-1 (PMC6288127; doi:10.1038/s41467-018-07649-1)
Supplement: Supplementary file 3 — Reporting Summary [file 41467_2018_7649_MOESM3_ESM.pdf]

nature research

Corresponding author(s): Bernhard O. Palsson

Reporting Summary

Nature Research wishes to improve the reproducibility of the work that we publish. This form provides structure for consistency and transparency in reporting. For further information on Nature Research policies, see Authors & Referees and the Editorial Policy Checklist.

Statistical parameters

When statistical analyses are reported, confirm that the following items are present in the relevant location (e.g. figure legend, table legend, main text, or Methods section).

n/a	Confirmed	
☐	☒	The exact sample size (n) for each experimental group/condition, given as a discrete number and unit of measurement
☒	☐	An indication of whether measurements were taken from distinct samples or whether the same sample was measured repeatedly
☐	☒	The statistical test(s) used AND whether they are one- or two-sided<br>*Only common tests should be described solely by name; describe more complex techniques in the Methods section.*
☐	☒	A description of all covariates tested
☒	☐	A description of any assumptions or corrections, such as tests of normality and adjustment for multiple comparisons
☐	☒	A full description of the statistics including central tendency (e.g. means) or other basic estimates (e.g. regression coefficient) AND variation (e.g. standard deviation) or associated estimates of uncertainty (e.g. confidence intervals)
☒	☐	For null hypothesis testing, the test statistic (e.g. F, t, r) with confidence intervals, effect sizes, degrees of freedom and P value noted<br>*Give P values as exact values whenever suitable.*
☒	☐	For Bayesian analysis, information on the choice of priors and Markov chain Monte Carlo settings
☒	☐	For hierarchical and complex designs, identification of the appropriate level for tests and full reporting of outcomes
☐	☒	Estimates of effect sizes (e.g. Cohen's d, Pearson's r), indicating how they were calculated
☐	☒	Clearly defined error bars<br>*State explicitly what error bars represent (e.g. SD, SE, CI)*

Our web collection on statistics for biologists may be useful.

Software and code

Policy information about availability of computer code

Data collection	No software was used
Data analysis	Simulation and data analysis was conducted in the R environment for statistical computing (version 3.3.3). Linear programming problems were constructed using the R packages sybil (version 2.0.0) and sybilccFBA (version 2.0.0) and solved using IBM CPLEX version 12.7.

For manuscripts utilizing custom algorithms or software that are central to the research but not yet described in published literature, software must be made available to editors/reviewers upon request. We strongly encourage code deposition in a community repository (e.g. GitHub). See the Nature Research guidelines for submitting code & software for further information.

Data

Policy information about availability of data

All manuscripts must include a data availability statement. This statement should provide the following information, where applicable:

- Accession codes, unique identifiers, or web links for publicly available datasets
- A list of figures that have associated raw data
- A description of any restrictions on data availability

Predicted kcat endpoints that are presented in this study are available from the authors upon request.

Field-specific reporting

Please select the best fit for your research. If you are not sure, read the appropriate sections before making your selection.

☒ Life sciences ☐ Behavioural & social sciences ☐ Ecological, evolutionary & environmental sciences

For a reference copy of the document with all sections, see nature.com/authors/policies/ReportingSummary-flat.pdf

Life sciences study design

All studies must disclose on these points even when the disclosure is negative.

Sample size	Sample sizes were subject to data availability in databases and publications. Described on page 7 and in the legend of Figure 4
Data exclusions	Published data sets were use for validation of our predictions. As described in the legend of Figure 4, simulated data is only shown for non-constrained reactions that contribute to growth. Constrained reactions were not compared as their kcat is pre-determined by our assumptions about constraints and would bias comparisons. Reactions that do not contribute to growth will never carry flux and are thus not adequately represented by MOMENT simulations that optimize for growth.
Replication	Simulations were replicated using different random seeds. Number of replicates are given in figure legends.
Randomization	Does not apply, as we use all available literature data on in vitro and in vivo turnover rates.
Blinding	Does not apply, as we use all available literature data on in vitro and in vivo turnover rates .

Reporting for specific materials, systems and methods

Materials & experimental systems

n/a	Involved in the study	
☒	☐	Unique biological materials
☒	☐	Antibodies
☒	☐	Eukaryotic cell lines
☒	☐	Palaeontology
☒	☐	Animals and other organisms
☒	☐	Human research participants

Methods

n/a	Involved in the study	
☒	☐	ChIP-seq
☒	☐	Flow cytometry
☒	☐	MRI-based neuroimaging
